# Microalbuminuria as an Independent Risk Factor for Developing Concentric Left Ventricular Hypertrophy in Primary Hypertension: A Single-Center Observational Study From South India

**DOI:** 10.7759/cureus.21119

**Published:** 2022-01-11

**Authors:** Sayyid Moidu, Akash T Oomen, Gopalakrishna Pillai, Sheejamol VS

**Affiliations:** 1 Internal Medicine, Amrita Institute of Medical Sciences and Research Centre, Kochi, IND; 2 Biostatistics, Amrita Institute of Medical Sciences and Research Centre, Kochi, IND

**Keywords:** concentric lvh, hypertension, primary hypertension, left ventricular hypertrophy, microalbuminuria

## Abstract

Introduction

Microalbuminuria and concentric left ventricular hypertrophy (LVH) are both associated with primary hypertension. We aimed to study the correlation between these two parameters in a cohort of patients with primary hypertension.

Methods

We conducted a single-center observational comparative study involving patients suffering from primary hypertension in a tertiary care hospital in the southern state of Kerala in India. Patients aged more than 18 years who were diagnosed to have primary hypertension were enrolled in the study irrespective of duration of illness or treatment status. The primary objective of the study was to assess whether microalbuminuria was an independent risk factor for concentric LVH in patients with primary hypertension. The secondary objective of the study was to study the relationship between various other studied biomarkers with concentric LVH in patients with primary hypertension.

Results

Microalbuminuria was found to be associated with concentric LVH in patients with primary hypertension (p=0.003). Multivariate regression analysis showed that serum creatinine, high diastolic blood pressure, and microalbuminuria appeared to be independent risk factors for concentric LVH (p<0.001, 0.016, and 0.016 respectively).

Conclusions

Microalbuminuria is a reliable marker for predicting the prevalence of concentric LVH in patients with primary hypertension. A high serum creatinine and high diastolic blood pressure are also independent risk factors for having concentric LVH.

## Introduction

Primary hypertension is one of the leading causes of heart disease and stroke in the world and it is no different in India. Epidemiological studies estimate that more than 200 million people suffer from primary hypertension in India alone and this prevalence seems to be increasing [1]. Other estimates suggest that hypertension directly contributes to around 57% of all deaths related to cerebrovascular accidents (CVA) and to around 24% of all deaths related to coronary artery disease (CAD) in India [2]. Hypertension is also the most common preventable risk factor in the development of cardiovascular disease, chronic kidney disease, in cognitive impairment, and is also the highest contributor to all-cause death as well as disability globally [3]. The correlation between the development of heart and kidney manifestations in primary hypertension remains unclear.

Microalbuminuria is defined as urinary albumin to creatinine ratio between 30mg/g to <300 mg/g [4]. Microalbuminuria is also well recognized as a risk factor for morbidity and mortality in patients with hypertension. It is a relatively early manifestation of renal damage which occurs secondary to hypertension and is a predictor for CAD and all cause-mortality in hypertensives [4]. Hence, we aimed to study the role of microalbuminuria as an independent risk factor for concentric left ventricular hypertrophy (LVH) in patients with primary hypertension.

## Materials and methods

Study design

We conducted a single-center observational comparative study involving patients suffering from primary hypertension in a tertiary care hospital in the southern state of Kerala in India over the course of one year between July 2020 to July 2021. Based on a previous paper by Sharma et al. where they observed a prevalence of 61.2% primary hypertension patients with LVH having microalbuminuria and 32.5% of primary hypertension patients without LVH having microalbuminuria, with a confidence interval of 95% and a margin of error of 20%, the minimum sample size required in each group was calculated to be 46 patients [5]. Hence 95 patients were enrolled in the study; 47 primary hypertension patients with concentric LVH and 48 primary hypertension patients without concentric LVH. The primary objective of the study was to assess whether microalbuminuria was an independent risk factor for concentric LVH in patients with primary hypertension. The secondary objective of the study was to study the relationship between various other studied biomarkers with concentric LVH in patients with primary hypertension. This study was approved by the institutional ethical committee (IRB-AIMS-2020-194). 

Inclusion and exclusion criteria

Patients aged more than 18 years who were diagnosed to have primary hypertension were enrolled in the study irrespective of the duration of illness or treatment status. Patients were excluded from the study if there was evidence of secondary hypertension due to any cause, peripheral vascular occlusive disease, coronary artery disease, any heart failure or arrhythmia, co-existing diabetes mellitus, history of a CVA, or if there was a history of any kidney disease which included previously identified overt proteinuria.

Data collection

Patients who were newly diagnosed to have primary hypertension or who had a previous diagnosis of primary hypertension who visited the internal medicine outpatient department were enrolled in the study after obtaining informed consent. The study consisted of 47 consecutive patients with concentric LVH and 48 consecutive patients without concentric LVH who attended the outpatient department. Patient demographics including age and gender were collected. The smoking status was ascertained. The blood pressure readings were taken once again after inclusion into the study just before obtaining baseline blood investigations. Each of these patients then underwent a set of investigations which included serum creatinine, fasting lipid profile, echocardiogram, and urine protein: creatinine ratio (PCR). This data was collected by the lead investigator and entered into a Microsoft Excel 2016 (Microsoft Corporation, Albuquerque, New Mexico, United States) master chart. Once an adequate sample size was achieved, this data was statistically analyzed. 

Definitions

Hypertension was defined as systolic blood pressure (SBP) ≥140 mmHg and/or a diastolic blood pressure (DBP) ≥90 mmHg taken on two separate occasions at least 24 hours apart [6]. Primary hypertension was defined as hypertension with no secondary etiological cause identified [6]. Microalbuminuria was defined as urinary albumin to creatinine ratio between 30mg/g to <300 mg/g in a 24-hour urine sample for which a spot protein: creatinine ratio surrogate was used [4]. Concentric LVH was diagnosed using echocardiography and was defined as an increase in the left ventricular (LV) mass. The LV mass was calculated using the formula [5]:

LV mass = 0.8 (1.04 ([LVIDd + PWTd + IVSTd] 3 - [LVIDd] 3))+ 0.6 g

where LVID is the the left ventricular internal dimension, VST is the interventricular septal thickness, and PWT is the posterior wall thickness.

Normal LV mass indexed to the body’s surface area was taken as 70 ± 9 g/m2 in males and 61 ± 8 g/m2 in females [7]. 

Statistical analysis

Statistical analysis was done using the IBM SPSS Statistics 20 Windows (SPSS Inc., Chicago, USA). Categorical variables were expressed as frequencies and percentages while continuous variables are expressed in terms of mean and standard deviation. Unpaired t-test was used to test the study the correlation between means of unrelated parameters if the data were normally distributed, while the Mann-Whitney U test was used in the case of non-normal distribution. The Chi-square test was used to study the relationship between categorical variables in the study. A multivariate logistic regression analysis was performed to find the independent risk factors for concentric LVH and the corresponding odds ratio (OR) with 95% confidence intervals was obtained. A p-value of <0.05 was considered statistically significant. All tests of statistical significance were two-tailed.

## Results

A total of 95 patients who fulfilled the inclusion criteria were enrolled in this study with 47 patients having concentric LVH and 48 patients having no concentric LVH. The LV mass index (g/m2) among those with and without concentric LVH were 182.34±17.5 g/m2 and 101.46±11.7 g/m2 respectively. 

On comparison of genders between the groups, we observed that among 51 male patients, 25 (49%) had concentric LVH while among 44 female patients, 22 (50%) had concentric LVH. There was no statistically significant association found between gender and concentric LVH (p=0.924). The mean age among those with and without LVH were 63.98±6.7 and 62.98±4.8 years respectively (p=0.409). The SBP among those with and without LVH were 168.30±14.8 and 166.25±14.4 mmHg respectively (p=0.5). The DBP among those with and without LVH were 106.81±9.3 and 88.75±13.3 mmHg respectively (p<0.001). Among 35 smokers in the study, 22 (62.9%) had concentric LVH and among 60 non-smokers, 25 (41.7.0%) had concentric LVH. There was a statistically significant correlation for the habit of smoking between the two groups (p=0.046).

When looking at the distribution of microalbuminuria in the two groups, we found that among 44 patients with microalbuminuria, 29 (65.9%) patients had concentric LVH and in the 51 patients without microalbuminuria, 18 (35.3%) patients had evidence of concentric LVH with a statistically significant correlation identified between the presence of microalbuminuria and the presence of concentric LVH (p=0.003). The mean LV mass index in the microalbuminuria group was 159.8±38.9 g/m2 while the mean LV mass index in the non-microalbuminuria group was 125.6±40.7 g/m2 with a statistically significant association (p<0.001). The urine PCR among those with and without concentric LVH were 51.51±23.9 mg/g and 22.65±5.3 mg/g respectively. There was a statistically significant correlation between urine PCR and concentric LVH with significantly higher urine PCR found in patients with concentric LVH (p<0.001).

With respect to the serum creatinine levels between the groups, we found that among the patients with concentric LVH, the mean serum creatine was 0.98±0.19 mg/dL while in the group with no concentric LVH, the mean was 0.78±0.17 mg/dL with a statistically significant correlation identified between high serum creatinine and the presence of concentric LVH (p<0.001). On comparison of serum triglyceride (TG) between the groups, we found that the mean serum TG in the group with concentric LVH was 193.1±.28.9 mg/dL while it was 175.6±15.7 mg/dL in the non-LVH group with a statistically significant correlation seen with higher TG levels in patients with LVH (p<0.001). 

When comparing the serum high-density lipoprotein (HDL) between the groups, we observed that the mean HDL was 41.4±6.9 mg/dL in the patients having concentric LVH while in the group with no concentric LVH, the mean was 51.15±9.3 mg/dL with a statistically significant correlation identified between low HDL levels and the presence of concentric LVH (p<0.001). With respect to the serum low-density lipoprotein (LDL) between the groups, we found that the mean serum LDL in the group with concentric LVH was 125±.14.4 mg/dL while it was 121±13.7 mg/dL in the non-LVH group. There was no statistically significant correlation between LDL levels and LVH (p=0.166).

With respect to the serum total cholesterol (TC) between the groups, The TC among those with and without concentric LVH were 200.4±22.2 mg/dL and 190.9±19.3 mg/dL respectively. There was a statistically significant correlation between TC and concentric LVH with significantly higher TC levels found in patients with concentric LVH (p=0.029). The study parameters are summarized in Table 1. 

**Table 1:** Comparison of study parameters between the groups ^1 ^Expressed as mean±standard deviation, LVH: Left ventricular hypertrophy

Parameter	Concentric LVH group (n=47)	No LVH group (n-48)	P value
Male gender	25 (49%)	26 (51%)	0.924
Age (years) ^_1_^	63.98±6.7	62.98±4.8	0.409
Smoker	22 (62.9%)	13 (37.1%)	0.046
Systolic blood pressure (mmHg)^_1_^	168.30±14.8	166.25±14.4	0.5
Diastolic blood pressure (mmHg) ^_1_^	106.81±9.3	88.75±13.3	<0.001
Serum creatinine ^1^	0.98±0.19	0.78±0.17	<0.001
Total cholesterol ^1^	200.4±22.2	190.9±19.3	0.029
Serum triglyceride ^1^	193.1±.28.9	175.6±15.7	<0.001
Low density lipoprotein ^1^	125±.14.4	121±13.7	0.166
High density lipoprotein ^1^	41.4±6.9	51.15±9.3	<0.001
Microalbuminuria	29 (65.9%)	15 (34.1%)	0.003

Using a stepwise multivariate logistic regression analysis to identify independent risk factors for concentric LVH, we observed that DBP, urine ACR, serum creatinine, and serum TG were independent risk factors for concentric LVH (p<0.001, 0.037, 0.008, and 0.024 respectively) (Table 2). 

**Table 2:** Multivariate analysis results ACR: Albumin:creatinine ratio

Variables	Odds ratio	P value	Confidence Interval	
			lower	Upper
Urine ACR	4.275	0.037	1.091	1.239
Serum creatinine	128.343	0.008	13.498	4709.209
Serum triglyceride	1.051	0.024	1.007	1.097
Diastolic blood pressure	1.154	< 0.001	1.076	1.262

## Discussion

This was a single-center observational comparative study conducted a tertiary care hospital in South India that aimed to study the correlation between microalbuminuria and concentric LVH in patients with primary hypertension. The primary objective of the study was to assess whether microalbuminuria was an independent risk factor for concentric LVH in patients with primary hypertension. The secondary objective of the study was to study the relationship between various other studied biomarkers with concentric LVH in patients with primary hypertension.

We found that in our cohort, there was a correlation identified between the presence of microalbuminuria and concentric LVH. Microalbuminuria was seen in approximately half our patients, out of which more than two-thirds were in the concentric LVH group. This correlation was also found on the multivariate logistic regression analysis suggesting that even when adjusting for confounders, microalbuminuria appears to be an independent risk factor for concentric LVH in patients with primary hypertension. We also found the converse to be true with significantly higher LV mass seen in patients with microalbuminuria. The reason for this could be muti-factorial and various previous reports have put forward numerous hypotheses. Alteration in the hemodynamics as well as an increase in blood pressure could lead to early kidney damage in primary hypertension which manifests as microalbuminuria. This rise in BP and alteration in the hemodynamics could also be responsible for the remodeling of the LV architecture which manifests as concentric LVH [4]. Hence, both these processes seem to be the end results of the same underlying mechanism. Other mechanisms put forward include a theory that suggests that microalbuminuria is a marker of vascular endothelial dysfunction [8]. The resulting raised vascular permeability is thought to predispose to greater penetration of the atherogenic lipoprotein particles into the wall of the arteries. Microalbuminuria is also thought to cause an impairment of the large arteries mechanics in essential hypertension. These factors all lead to both structural and functional changes in the LV [8]. 

Interestingly, we also found a correlation between the diastolic BP and concentric LVH in this study with patients with LVH having almost 20mmHg higher mean DBP. This finding was replicated on the multivariate analysis with DBP found to be one of the independent risk factors for concentric LVH in primary hypertension. There have been reports of associations found between high DBP and microalbuminuria in hypertensives [9]. This means that a markedly and repeatedly elevated DBP could act as a non-invasive and easily reproducible marker for both microalbuminuria and concentric LVH in patients with primary hypertension. The other markers of concentric LVH in this study based on regression analysis were the serum creatinine and serum triglyceride levels. Microalbuminuria could indicate early renal damage, which could also be manifesting as elevated serum creatinine levels. Previous reports have suggested that LVH is a marker of renal disease in patients who are at high risk of developing cardiovascular disease [10]. Hence our findings add weight to this and prove that LVH and microalbuminuria have a complex interplay in hypertension. A few other biomarkers including smoking, total cholesterol levels, and serum HDL showed a statistical correlation with LVH but failed to be identified as independent risk factors on multiple regression. However, they may play a role and further studies are required to further assess their usefulness in predicting concentric LVH in primary hypertension. 

Our study had its strengths and its limitations. The strengths included the prospective nature of the study which allowed us to uniformly collect the necessary data from our patients including the standardization of the definitions of concentric LVH as well as methods to determine the values of the various biomarkers studied. The limitations include the single-center nature of our study. Our findings are not indicative of the general population because of this and further multi-center studies are warranted to reproduce these findings in the general population. Another limitation is the relatively small sample size. Even though the sample size was pre-calculated at the beginning of the study, it may be too underpowered to accurately reflect some of the biomarkers studied. Despite our best efforts, there could have been a degree of inter-technician variability based on the person performing the echocardiography, which could have influenced the LV thickness reading. Finally, we may not have accounted for all of the confounders in the study and this may have influenced the results.

## Conclusions

We found that microalbuminuria correlates with the presence of concentric LVH in patients with primary hypertension with a higher prevalence of microalbuminuria noted in patients with concentric LVH. In addition to microalbuminuria, high serum creatinine, high serum triglycerides, and high diastolic blood pressure were the other independent risk factors found to be associated with concentric LVH in this study.

Our study suggests that microalbuminuria is a relatively easy and non-invasive biomarker that could predict the involvement of LV hypertrophy in patients with primary hypertension. These appear to be the result of the same underlying pathology in hypertension and seem to be interlinked. From our findings, a high DBP or high serum creatinine could also be markers of heart involvement in primary LVH. These correlations warrant further research to establish the link in a more heterogeneous and larger population.
